# Breast Cancer—Epidemiology, Risk Factors, Classification, Prognostic Markers, and Current Treatment Strategies—An Updated Review

**DOI:** 10.3390/cancers13174287

**Published:** 2021-08-25

**Authors:** Sergiusz Łukasiewicz, Marcin Czeczelewski, Alicja Forma, Jacek Baj, Robert Sitarz, Andrzej Stanisławek

**Affiliations:** 1 Department of Surgical Oncology, Center of Oncology of the Lublin Region St. Jana z Dukli, 20-091 Lublin, Poland; Slukasiewicz@cozl.pl (S.Ł.); andrzej.stanislawek@umlub.pl (A.S.); 2 Department of Forensic Medicine, Medical University of Lublin, 20-090 Lublin, Poland; marcin.czeczelewski@gmail.com (M.C.); aforma@onet.pl (A.F.); 3 Department of Human Anatomy, Medical University of Lublin, 20-090 Lublin, Poland; jacek.baj@umlub.pl; 4 Department of Oncology, Chair of Oncology and Environmental Health, Medical University of Lublin, 20-081 Lublin, Poland

**Keywords:** breast cancer, epidemiology, risk factors, classification, diagnosis, prognosis, marker, treatment

## Abstract

**Simple Summary:**

Breast cancer is the most common cancer among women. It is estimated that 2.3 million new cases of BC are diagnosed globally each year. Based on mRNA gene expression levels, BC can be divided into molecular subtypes that provide insights into new treatment strategies and patient stratifications that impact the management of BC patients. This review addresses the overview on the BC epidemiology, risk factors, classification with an emphasis on molecular types, prognostic biomarkers, as well as possible treatment modalities.

**Abstract:**

Breast cancer (BC) is the most frequently diagnosed cancer in women worldwide with more than 2 million new cases in 2020. Its incidence and death rates have increased over the last three decades due to the change in risk factor profiles, better cancer registration, and cancer detection. The number of risk factors of BC is significant and includes both the modifiable factors and non-modifiable factors. Currently, about 80% of patients with BC are individuals aged >50. Survival depends on both stage and molecular subtype. Invasive BCs comprise wide spectrum tumors that show a variation concerning their clinical presentation, behavior, and morphology. Based on mRNA gene expression levels, BC can be divided into molecular subtypes (Luminal A, Luminal B, HER2-enriched, and basal-like). The molecular subtypes provide insights into new treatment strategies and patient stratifications that impact the management of BC patients. The eighth edition of TNM classification outlines a new staging system for BC that, in addition to anatomical features, acknowledges biological factors. Treatment of breast cancer is complex and involves a combination of different modalities including surgery, radiotherapy, chemotherapy, hormonal therapy, or biological therapies delivered in diverse sequences.

## 1. Introduction

Being characterized by six major hallmarks, carcinogenesis might occur in every cell, tissue, and organ, leading to the pathological alternations that result in a vast number of cancers. The major mechanisms that enable its progression include evasion of apoptosis, limitless capacity to divide, enhanced angiogenesis, resistance to anti-growth signals and induction of own growth signals, as well as the capacity to metastasize [1]. Carcinogenesis is a multifactorial process that is primarily stimulated by both—genetic predispositions and environmental causes. The number of cancer-related deaths is disturbingly increasing every year ranking them as one of the major causes of death worldwide. Even though a significant number of cancers do not always need to result in death, they significantly lower the quality of life and require larger costs in general.

Breast cancer is currently one of the most prevalently diagnosed cancers and the 5th cause of cancer-related deaths with an estimated number of 2.3 million new cases worldwide according to the GLOBOCAN 2020 data [2]. Deaths due to breast cancer are more prevalently reported (an incidence rate approximately 88% higher) in transitioning countries (Melanesia, Western Africa, Micronesia/Polynesia, and the Caribbean) compared to the transitioned ones (Australia/New Zealand, Western Europe, Northern America, and Northern Europe). Several procedures such as preventive behaviors in general as well as screening programs are crucial regarding a possible minimization of breast cancer incidence rate and the implementation of early treatment. Currently, it is the Breast Health Global Initiative (BHGI) that is responsible for the preparation of proper guidelines and the approaches to provide the most sufficient breast cancer control worldwide [3]. In this review article, we have focused on the female breast cancer specifically since as abovementioned, it currently constitutes the most prevalent cancer amongst females.

## 2. Breast Cancer Epidemiology

According to the WHO, malignant neoplasms are the greatest worldwide burden for women, estimated at 107.8 million Disability-Adjusted Life Years (DALYs), of which 19.6 million DALYs are due to breast cancer. [4]. Breast cancer is the most frequently diagnosed cancer in women worldwide with 2.26 million [95% UI, 2.24–2.79 million] new cases in 2020 [5]. In the United States, breast cancer alone is expected to account for 29% of all new cancers in women [6]. The 2018 GLOBOCAN data shows that age-standardized incidence rates (ASIR) of breast cancer are strongly and positively associated with the Human Development Index (HDI) [7]. According to 2020 data, the ASIR was the highest in very high HDI countries (75.6 per 100,000) while it was more than 200% lower in medium and low HDI countries (27.8 per 100,000 and 36.1 per 100,000 respectively) [5].

Besides being the most common, breast cancer is also the leading cause of cancer death in women worldwide. Globally, breast cancer was responsible for 684,996 deaths [95% UI, 675,493–694,633] at an age-adjusted rate of 13.6/100,000 [5]. Although incidence rates were the highest in developed regions, the countries in Asia and Africa shared 63% of total deaths in 2020 [5]. Most women who develop breast cancer in a high-income country will survive; the opposite is true for women in most low-income and many middle-income countries [8].

In 2020 breast cancer mortality-to-incidence ratio (MIR) as a representative indicator of 5-year survival rates [9] was 0.30 globally [5]. Taking into consideration the clinical extent of breast cancer, in locations with developed health care (Hong-Kong, Singapore, Turkey) the 5-year survival was 89.6% for localized and 75.4% for regional cancer. In less developed countries (Costa Rica, India, Philippines, Saudi Arabia, Thailand) the survival rates were 76.3% and 47.4% for localized and regional breast cancer respectively [10].

### Trends

Breast cancer incidence and death rates have increased over the last three decades. Between 1990 and 2016 breast cancer incidence has more than doubled in 60/102 countries (e.g., Afghanistan, Philippines, Brazil, Argentina), whereas deaths have doubled in 43/102 countries (e.g., Yemen, Paraguay, Libya, Saudi Arabia) [11]. Current projections indicate that by 2030 the worldwide number of new cases diagnosed reach 2.7 million annually, while the number of deaths 0.87 million [12]. In low- and medium-income countries, the breast cancer incidence is expected to increase further due to the westernization of lifestyles (e.g., delayed pregnancies, reduced breastfeeding, low age at menarche, lack of physical activity, and poor diet), better cancer registration, and cancer detection [13].

## 3. Risk Factors of Breast Cancer

The number of risk factors of breast cancer is significant and includes both modifiable factors and non-modifiable factors (Table 1).

### 3.1. Non-Modifiable Factors

#### 3.1.1. Female Sex

Female sex constitutes one of the major factors associated with an increased risk of breast cancer primarily because of the enhanced hormonal stimulation. Unlike men who present insignificant estrogen levels, women have breast cells which are very vulnerable to hormones (estrogen and progesterone in particular) as well as any disruptions in their balance. Circulating estrogens and androgens are positively associated with an increased risk of breast cancer [14]. The alternations within the physiological levels of the endogenous levels of sex hormones result in a higher risk of breast cancer in the case of premenopausal and postmenopausal women; these observations were also supported by the Endogenous Hormones and Breast Cancer Collaborative Group [15,16,17].

Less than 1% of all breast cancers occur in men. However, breast cancer in men is a rare disease that’s at the time of diagnosis tends to be more advanced than in women. The average age of men at the diagnosis is about 67. The important factors increase a man’s risk of breast cancer are: older age, BRCA2/BRCA1 mutations, increased estrogen levels, Klinefelter syndrome, family history of breast cancer, and radiation exposure [18].

#### 3.1.2. Older Age

Currently, about 80% of patients with breast cancer are individuals aged >50 while at the same time more than 40% are those more than 65 years old [19,20,21]. The risk of developing breast cancer increases as follows—the 1.5% risk at age 40, 3% at age 50, and more than 4% at age 70 [22]. Interestingly, a relationship between a particular molecular subtype of cancer and a patient’s age was observed –aggressive resistant triple-negative breast cancer subtype is most commonly diagnosed in groups under 40 age, while in patients >70, it is luminal A subtype [21]. Generally, the occurrence of cancer in older age is not only limited to breast cancer; the accumulation of a vast number of cellular alternations and exposition to potential carcinogens results in an increase of carcinogenesis with time.

#### 3.1.3. Family History

A family history of breast cancer constitutes a major factor significantly associated with an increased risk of breast cancer. Approximately 13–19% of patients diagnosed with breast cancer report a first-degree relative affected by the same condition [23]. Besides, the risk of breast cancer significantly increases with an increasing number of first-degree relatives affected; the risk might be even higher when the affected relatives are under 50 years old [24,25,26]. The incidence rate of breast cancer is significantly higher in all of the patients with a family history despite the age. This association is driven by epigenetic changes as well as environmental factors acting as potential triggers [27]. A family history of ovarian cancer—especially those characterized by *BRCA1* and *BRCA2* mutations—might also induce a greater risk of breast cancer [28].

#### 3.1.4. Genetic Mutations

Several genetic mutations were reported to be highly associated with an increased risk of breast cancer. Two major genes characterized by a high penetrance are *BRCA1* (located on chromosome 17) and *BRCA2* (located on chromosome 13). They are primarily linked to the increased risk of breast carcinogenesis [29]. The mutations within the above-mentioned genes are mainly inherited in an autosomal dominant manner, however, sporadic mutations are also commonly reported. Other highly penetrant breast cancer genes include *TP53*, *CDH1*, *PTEN*, and *STK11* [30,31,32,33,34]. Except for the increased risk of breast cancer, carriers of such mutations are more susceptible to ovarian cancer as well. A significant number of DNA repair genes that can interact with *BRCA* genes including *ATM*, *PALB2*, *BRIP1*, or *CHEK2*, were reported to be involved in the induction of breast carcinogenesis; those are however characterized by a lower penetrance (moderate degree) compared to *BRCA1* or *BRCA2* (Table 2) [29,35,36,37,38]. According to quite recent Polish research, mutations within the *XRCC2* gene could also be potentially associated with an increased risk of breast cancer [39].

#### 3.1.5. Race/Ethnicity

Disparities regarding race and ethnicity remain widely observed among individuals affected by breast cancer; the mechanisms associated with this phenomenon are not yet understood. Generally, the breast cancer incidence rate remains the highest among white non-Hispanic women [51,52]. Contrarily, the mortality rate due to this malignancy is significantly higher among black women; this group is also characterized by the lowest survival rates [53].

#### 3.1.6. Reproductive History

Numerous studies confirmed a strict relationship between exposure to endogenous hormones—estrogen and progesterone in particular—and excessive risk of breast cancer in females. Therefore, the occurrence of specific events such as pregnancy, breastfeeding, first menstruation, and menopause along with their duration and the concomitant hormonal imbalance, are crucial in terms of a potential induction of the carcinogenic events in the breast microenvironment. The first full-term pregnancy at an early age (especially in the early twenties) along with a subsequently increasing number of births are associated with a reduced risk of breast cancer [54,55]. Besides, the pregnancy itself provides protective effects against potential cancer. However, protection was observed at approximately the 34th pregnancy week and was not confirmed for the pregnancies lasting for 33 weeks or less [56]. Women with a history of preeclampsia during pregnancy or children born to a preeclamptic pregnancy are at lower risk of developing breast cancer [57]. No association between the increased breast cancer risk and abortion was stated so far [58].

The dysregulated hormone levels during preeclampsia including increased progesterone and reduced estrogen levels along with insulin, cortisol, insulin-like growth factor-1, androgens, human chorionic gonadotropin, corticotropin-releasing factor, and IGF-1 binding protein deviating from the physiological ranges, show a protective effect preventing from breast carcinogenesis. The longer duration of the breastfeeding period also reduces the risk of both the ER/PR-positive and -negative cancers [59]. Early age at menarche is another risk factor of breast cancer; it is possibly also associated with a tumor grade and lymph node involvement [60]. Besides, the earlier age of the first menstruation could result in an overall poorer prognosis. Contrarily, early menopause despite whether natural or surgical, lowers the breast cancer risk [61].

#### 3.1.7. Density of Breast Tissue

The density of breast tissue remains inconsistent throughout the lifetime; however, several categories including low-density, high-density, and fatty breasts have been established in clinical practice. Greater density of breasts is observed in females of younger age and lower BMI, who are pregnant or during the breastfeeding period, as well as during the intake of hormonal replacement therapy [62]. Generally, the greater breast tissue density correlates with the greater breast cancer risk; this trend is observed both in premenopausal and postmenopausal females [63]. It was proposed that screening of breast tissue density could be a promising, non-invasive, and quick method enabling rational surveillance of females at increased risk of cancer [64].

#### 3.1.8. History of Breast Cancer and Benign Breast Diseases

Personal history of breast cancer is associated with a greater risk of a renewed cancerous lesions within the breasts [65]. Besides, a history of any other non-cancerous alternations in breasts such as atypical hyperplasia, carcinoma in situ, or many other proliferative or non-proliferative lesions, also increases the risk significantly [66,67,68]. The histologic classification of benign lesions and a family history of breast cancer are two factors that are strongly associated with breast cancer risk [66].

#### 3.1.9. Previous Radiation Therapy

The risk of secondary malignancies after radiotherapy treatment remains an individual matter that depends on the patient’s characteristics, even though it is a quite frequent phenomenon that arises much clinical concern. Cancer induced by radiation therapy is strictly associated with an individual’s age; patients who receive radiation therapy before the age of 30, are at a greater risk of breast cancer [69]. The selection of proper radiotherapy technique is crucial in terms of secondary cancer risk—for instance, tangential field IMRT (2F-IMRT) is associated with a significantly lower risk compared to multiple-field IMRT (6F-IMRT) or double partial arcs (VMAT) [70]. Besides, the family history of breast cancer in patients who receive radiotherapy additionally enhances the risk of cancer occurrence [71]. However, Bartelink et al. showed that additional radiation (16 Gy) to the tumor bed combined with standard radiotherapy might decrease the risk of local recurrence [72].

### 3.2. Modifiable Factors

#### 3.2.1. Chosen Drugs

Data from some research indicates that the intake of diethylstilbestrol during pregnancy might be associated with a greater risk of breast cancer in children; this, however, remains inconsistent between studies and requires further evaluation [73,74]. The intake of diethylstilbestrol during pregnancy is associated with an increased risk of breast cancer not only in mothers but also in the offspring [75]. This relationship is observed despite the expression of neither estrogen nor progesterone receptors and might be associated with every breast cancer histological type. The risk increases with age; women at age of ≥40 years are nearly 1.9 times more susceptible compared to women under 40. Moreover, breast cancer risk increases with greater diethylstilbestrol doses [76]. Numerous researches indicate that females who use hormonal replacement therapy (HRT) especially longer than 5 or 7 years are also at increased risk of breast cancer [77,78]. Several studies indicated that the intake of chosen antidepressants, mainly paroxetine, tricyclic antidepressants, and selective serotonin reuptake inhibitors might be associated with a greater risk of breast cancer [79,80]. Lawlor et al. showed that similar risk might be achieved due to the prolonged intake of antibiotics; Friedman et al. observed that breast risk is mostly elevated while using tetracyclines [81,82]. Attempts were made to investigate a potential relationship between hypertensive medications, non-steroidal anti-inflammatory drugs, as well as statins, and an elevated risk of breast cancer, however, this data remains highly inconsistent [83,84,85].

#### 3.2.2. Physical Activity

Even though the mechanism remains yet undeciphered, regular physical activity is considered to be a protective factor of breast cancer incidence [86,87]. Chen et al. observed that amongst females with a family history of breast cancer, physical activity was associated with a reduced risk of cancer but limited only to the postmenopausal period [88]. However, physical activity is beneficial not only in females with a family history of breast cancer but also in those without such a history. Contrarily to the above-mentioned study, Thune et al. pointed out more pronounced effects in premenopausal females [89]. There are several hypotheses aiming to explain the protective role of physical activity in terms of breast cancer incidence; physical activity might prevent cancer by reducing the exposure to the endogenous sex hormones, altering immune system responses or insulin-like growth factor-1 levels [88,90,91].

#### 3.2.3. Body Mass Index

According to epidemiological evidence, obesity is associated with a greater probability of breast cancer. This association is mostly intensified in obese post-menopausal females who tend to develop estrogen-receptor-positive breast cancer. Yet, independently to menopausal status, obese women achieve poorer clinical outcomes [92]. Wang et al. showed that females above 50 years old with greater Body Mass Index (BMI) are at a greater risk of cancer compared to those with low BMI [93]. Besides, the researchers observed that greater BMI is associated with more aggressive biological features of tumor including a higher percentage of lymph node metastasis and greater size. Obesity might be a reason for greater mortality rates and a higher probability of cancer relapse, especially in premenopausal women [94]. Increased body fat might enhance the inflammatory state and affects the levels of circulating hormones facilitating pro-carcinogenic events [95]. Thus, poorer clinical outcomes are primarily observed in females with BMI ≥ 25 kg/m^2^ [96]. Interestingly, postmenopausal women tend to present poorer clinical outcomes despite proper BMI values but namely due to excessive fat volume [97]. Greater breast cancer risk with regards to BMI also correlates with the concomitant family history of breast cancer [98].

#### 3.2.4. Alcohol Intake

Numerous evidences confirm that excessive alcohol consumption is a factor that might enhance the risk of malignancies within the gastrointestinal tract; however, it was proved that it is also linked to the risk of breast cancer. Namely, it is not alcohol type but rather the content of alcoholic beverages that mostly affect the risk of cancer. The explanation for this association is the increased levels of estrogens induced by the alcohol intake and thus hormonal imbalance affecting the risk of carcinogenesis within the female organs [99,100]. Besides, alcohol intake often results in excessive fat gain with higher BMI levels, which additionally increases the risk. Other hypotheses include direct and indirect carcinogenic effects of alcohol metabolites and alcohol-related impaired nutrient intake [101]. Alcohol consumption was observed to increase the risk of estrogen-positive breast cancers in particular [102]. Consumed before the first pregnancy, it significantly contributes to the induction of morphological alterations of breast tissue, predisposing it to further carcinogenic events [103].

#### 3.2.5. Smoking

Carcinogens found in tobacco are transported to the breast tissue increasing the plausibility of mutations within oncogenes and suppressor genes (*p53* in particular). Thus, not only active but also passive smoking significantly contributes to the induction of pro-carcinogenic events [104]. Besides, longer smoking history, as well as smoking before the first full-term pregnancy, are additional risk factors that are additionally pronounced in females with a family history of breast cancer [105,106,107,108].

#### 3.2.6. Insufficient Vitamin Supplementation

Vitamins exert anticancer properties, which might potentially benefit in the prevention of several malignancies including breast cancer, however, the mechanism is not yet fully understood. Attempts are continually made to analyze the effects of vitamin intake (vitamin C, vitamin E, B-group vitamins, folic acid, multivitamin) on the risk of breast cancer, nevertheless, the data remains inconsistent and not sufficient to compare the results and draw credible data [108]. In terms of breast cancer, most studies are currently focused on vitamin D supplementation confirming its potentially protective effects [109,110,111]. High serum 25-hydroxyvitamin D levels are associated with a lower incidence rate of breast cancer in premenopausal and postmenopausal women [110,112]. Intensified expression of vitamin D receptors was shown to be associated with lower mortality rates due to breast cancer [113]. Even so, further evaluation is required since data remains inconsistent in this matter [108,114].

#### 3.2.7. Exposure to Artificial Light

Artificial light at night (ALAN) has been recently linked to increased breast cancer risk. The probable causation might be a disrupted melatonin rhythm and subsequent epigenetic alterations [115]. According to the studies conducted so far, increased exposure to ALAN is associated with a significantly greater risk of breast cancer compared to individuals with lowered ALAN exposure [116]. Nonetheless, data regarding the excessive usage of LED electronic devices and increased risk of breast cancer is insufficient and requires further evaluation as some results are contradictory [116].

#### 3.2.8. Intake of Processed Food/Diet

According to the World Health Organization (WHO), highly processed meat was classified as a Group 1 carcinogen that might increase the risk of not only gastrointestinal malignancies but also breast cancer. Similar observations were made in terms of an excessive intake of saturated fats [117]. Ultra-processed food is rich in sodium, fat, and sugar which subsequently predisposes to obesity recognized as another factor of breast cancer risk [118]. It was observed that a 10% increase of ultra-processed food in the diet is associated with an 11% greater risk of breast cancer [118]. Contrarily, a diet high in vegetables, fruits, legumes, whole grains, and lean protein is associated with a lowered risk of breast cancer [119]. Generally, a diet that includes food containing high amounts of n-3 PUFA, vitamin D, fiber, folate, and phytoestrogen might be beneficial as a prevention of breast cancer [120]. Besides, lower intake of n-6 PUFA and saturated fat is recommended. Several in vitro and in vivo studies also suggest that specific compounds found in green tea might present anti-cancer effects which has also been studied regarding breast cancer [121]. Similar properties were observed in case of turmeric-derived curcuminoids as well as sulforaphane (SFN) [122,123].

#### 3.2.9. Exposure to Chemical

Chronic exposure to chemicals can promote breast carcinogenesis by affecting the tumor microenvironment subsequently inducing epigenetic alterations along with the induction of pro-carcinogenic events [124]. Females chronically exposed to chemicals present significantly greater plausibility of breast cancer which is further positively associated with the duration of the exposure [125]. The number of chemicals proposed to induce breast carcinogenesis is significant; so far, dichlorodiphenyltrichloroethane (DDT) and polychlorinated biphenyl (PCB) are mostly investigated in terms of breast cancer since early exposure to those chemicals disrupts the development of mammary glands [126,127]. A potential relationship was also observed in the case of increased exposure to polycyclic aromatic hydrocarbons (PAH), synthetic fibers, organic solvents, oil mist, and insecticides [128].

#### 3.2.10. Other Drugs

Other drugs that might constitute potential risk factors for breast cancer include antibiotics, antidepressants, statins, antihypertensive medications (e.g., calcium channel blockers, angiotensin II-converting enzyme inhibitors), as well as NSAIDs (including aspirin, ibuprofen) [129,130,131,132,133].

## 4. Breast Cancer Classification

### 4.1. Histological Classification

Invasive breast cancers (IBC) comprise wide spectrum tumors that show a variation concerning their clinical presentation, behavior, and morphology. The World Health Organization (WHO) distinguish at least 18 different histological breast cancer types [134].

Invasive breast cancer of no special type (NST), formerly known as invasive ductal carcinoma is the most frequent subgroup (40–80%) [135]. This type is diagnosed by default as a tumor that fails to be classified into one of the histological special types [134]. About 25% of invasive breast cancers present distinctive growth patterns and cytological features, hence, they are recognized as specific subtypes (e.g., invasive lobular carcinoma, tubular, mucinous A, mucinous B, neuroendocrine) [136].

Molecular classification independently from histological subtypes, invasive breast cancer can be divided into molecular subtypes based on mRNA gene expression levels. In 2000, Perou et al. on a sample of 38 breast cancers identified 4 molecular subtypes from microarray gene expression data: Luminal, HER2-enriched, Basal-like, and Normal Breast-like [137]. Further studies allowed to divide the Luminal group into two subgroups (Luminal A and B) [138,139]. The normal breast-like subtype has subsequently been omitted, as it is thought to represent sample contamination by normal mammary glands. In the Cancer Genome Atlas Project (TCGA) over 300 primary tumors were thoroughly profiled (at DNA, RNA, and protein levels) and combined in biological homogenous groups of tumors. The consensus clustering confirmed the distinction of four main breast cancer intrinsic subtypes based on mRNA gene expression levels only (Luminal A, Luminal B, HER2-enriched, and basal-like) [140]. Additionally, the 5th intrinsic subtype—claudin-low breast cancer was discovered in 2007 in an integrated analysis of human and murine mammary tumors [141].

In 2009, Parker et al. developed a 50-gene signature for subtype assignment, known as PAM50, that could reliably classify particular breast cancer into the main intrinsic subtypes with 93% accuracy [142]. PAM50 is now clinically implemented worldwide using the NanoString nCounter^®^, which is the basis for the Prosigna^®^ test. The Prosigna^®^ combines the PAM50 assay as well as clinical information to assess the risk of distant relapse estimation in postmenopausal women with hormone receptor-positive, node-negative, or node-positive early-stage breast cancer patients, and is a daily-used tool assessing the indication of adjuvant chemotherapy [143,144,145].

### 4.2. Luminal Breast Cancer

Luminal breast cancers are ER-positive tumors that comprise almost 70% of all cases of breast cancers in Western populations [146]. Most commonly Luminal-like cancers present as IBC of no special subtype, but they may infrequently differentiate into invasive lobular, tubular, invasive cribriform, mucinous, and invasive micropapillary carcinomas [147,148]. Two main biological processes: proliferation-related pathways and luminal-regulated pathways distinguish Luminal-like tumors into Luminal A and B subtypes with different clinical outcomes.

Luminal A tumors are characterized by presence of estrogen-receptor (ER) and/or progesterone-receptor (PR) and absence of HER2. In this subtype the ER transcription factors activate genes, the expression of which is characteristic for luminal epithelium lining the mammary ducts [149,150]. It also presents a low expression of genes related to cell proliferation [151]. Clinically they are low-grade, slow-growing, and tend to have the best prognosis.

In contrast to subtype A, Luminal B tumors are higher grade and has worse prognosis. They are ER positive and may be PR negative and/or HER2 positive. Additionally, it has high expression of proliferation-related genes (e.g., MKI67 and AURKA) [152,153,154]. This subtype has lower expression of genes or proteins typical for luminal epithelium such as the PR [150,155] and FOXA1 [146,156], but not the ER [157]. ER is similarly expressed in both A and B subtypes and is used to distinguish luminal from non-luminal disease.

### 4.3. HER2-Enriched Breast Cancer

The HER2-enriched group makes up 10–15% of breast cancers. It is characterized by the high expression of the HER2 with the absence of ER and PR. This subtype mainly expresses proliferation—related genes and proteins (e.g., ERBB2/HER2 and GRB7), rather than luminal and basal gene and protein clusters [154,156,157]. Additionally, in the HER2-enriched subtype there is evidence of mutagenesis mediated by APOBEC3B. APOBEC3B is a subclass of APOBEC cytidine deaminases, which induce cytosine mutation biases and is a source of mutation clusters [158,159,160].

HER2-enriched cancers grow faster than luminal cancers and used to have the worst prognosis of subtypes before the introduction of HER2-targeted therapies. Importantly, the HER2-enriched subtype is not synonymous with clinically HER2-positive breast cancer because many ER-positive/HER2-positive tumors qualify for the luminal B group. Moreover, about 30% of HER2-enriched tumors are classified as clinically HER2-negative based on immunohistochemistry (IHC) and/or fluorescence in situ hybridization (FISH) methods [161].

### 4.4. Basal-Like/Triple-Negative Breast Cancer

The Triple-Negative Breast Cancer (TNBC) is a heterogeneous collection of breast cancers characterized as ER-negative, PR-negative, and HER2-negative. They constitute about 20% of all breast cancers. TNBC is more common among women younger than 40 years of age and African-American women [161]. The majority (approximately 80%) of breast cancers arising in BRCA1 germline mutation are TNBC, while 11–16% of all TNBC harbor BRCA1 or BRCA2 germline mutations. TNBC tends to be biologically aggressive and is often associated with a worse prognosis [162]. The most common histology seen in TNBC is infiltrating ductal carcinoma, but it may also present as medullary-like cancers with a prominent lymphocytic infiltrate; metaplastic cancers, which may show squamous or spindle cell differentiation; and rare special type cancers like adenoid cystic carcinoma (AdCC) [163,164,165].

The terms basal-like and TNBC have been used interchangeably; however, not all TNBC are of the basal type. On gene expression profiling, TNBCs can be subdivided into six subtypes: basal-like (BL1 and BL2), mesenchymal (M), mesenchymal stem-like (MSL), immunomodulatory (IM), and luminal androgen receptor (LAR), as well as an unspecified group (UNS) [166,167]. However, the clinical relevance of the subtyping still unclear, and more research is needed to clarify its impact on TNBC treatment decisions [168].

### 4.5. Claudin-Low Breast Cancer

Claudin-low (CL) breast cancers are poor prognosis tumors being mostly ER-negative, PR-negative, and HER2-negative. CL tumors account for 7–14% of all invasive breast cancers [147]. No differences in survival rates were observed between claudin-low tumors and other poor-prognosis subtypes (Luminal B, HER2-enriched, and Basal-like). CL subtype is characterized by the low expression of genes involved in cell-cell adhesion, including claudins 3, 4, and 7, occludin, and E-cadherin. Besides, these tumors show high expression of epithelial-mesenchymal transition (EMT) genes and stem cell-like gene expression patterns [169,170]. Moreover, CL tumors have marked immune and stromal cell infiltration [171]. Due to their less differentiated state and a preventive effect of the EMT-related transcription factor, ZEB1 CL tumors are often genomically stable [172,173].

### 4.6. Surrogate Markers Classification

In clinical practice, the key question is the discrimination between patients who will or will not benefit from particular therapies. By using molecular assays, more patients can be spared adjuvant chemotherapy, but these tests are associated with significant costs. Therefore, surrogate subgroups based on pathological morphology and widely available immunohistochemical (IHC) markers are used as a tool for risk stratification and guidance of adjuvant therapy [174]. A combination of the routine pathological markers ER, PR, and HER2 is used to classify tumors into intrinsic subtypes [175]. Semiquantitative evaluation of Ki-67 and PR is helpful for further typing of the Luminal subtype [176,177]. Moreover, evaluation of cytokeratin 5/6 and epidermal growth factor receptor is utilized to identify the Basal-like breast cancer among the TNBC [178].

In St. Gallen’s 2013 guidelines the IHC-based surrogate subtype classification was recommended for clinical decision making [179]. However, these IHC-based markers are only a surrogate and cannot establish the intrinsic subtype of any given cancer, with discordance rates between IHC-based markers and gene-based assays as high as 30% [180].

### 4.7. American Joint Committee on Cancer Classification

The baseline tool to estimate the likely prognosis of patients with breast cancer is the AJCC staging system that includes grading, immunohistochemistry biomarkers, and anatomical advancement of the disease. Since its inception in 1977, the American Joint Committee on Cancer (AJCC) has published an internationally accepted staging system based on anatomic findings: tumor size (T), nodal status (N), and metastases (M). However, gene expression profiling has identified several molecular subtypes of breast cancer [181]. The eighth edition of the AJCC staging manual (2018), outlines a new prognostic staging system for breast cancer that, in addition to anatomical features, acknowledges biological factors [182]. These factors—ER, PR, HER2, grade, and multigene assays—are recommended in practice to define prognosis [183,184].

The most widely used histologic grading system of breast cancer is the Elston-Ellis modification [185] of Scarff-Bloom-Richardson grading system [186], also known as the Nottingham grading system. The grade of a tumor is determined by assessing morphologic features: (a) formation of tubules, (b) mitotic count, (c) variability, and the size and shape of cellular nuclei. A score between 1 (most favorable) and 3 (least favorable) is assigned for each feature. Grade 1 corresponds to combined scores between 3 and 5, grade 2 corresponds to a combined score of 6 or 7, and grade 3 corresponds to a combined score of 8 or 9.

In addition to grading and biomarkers, the commercially available multigene assays provide additional prognostic information suitable for incorporation in the AJCC 8th edition. The 21-gene assay Oncotype DX^®^ assessed by reverse transcription-polymerase chain reaction (RT-PCR) was the only assay sufficiently evaluated and included in the staging system. This assay is valuable in the staging of patients with hormone receptor-positive, HER2-negative, node-negative tumors that are <5 cm. Patients with results of the assay (Recurrence Score) less than 11 had excellent disease-free survival at 6.9 years of 98.6% with endocrine therapy alone [187]. Hence, adjuvant systemic chemotherapy can be safely omitted in patients with a low-risk multigene assay [188].

The AJCC staging manual includes a pathological and a clinical-stage group. The clinical prognostic stage group should be utilized in all patients on initial evaluation before any systemic therapy. Clinical staging uses the TNM anatomical information, grading, and expression of these three biomarkers. When patients undergo surgical resection of their primary tumor, the post-resection anatomic information coupled with the pretreatment biomarker findings results in the final Pathologic Prognostic Stage Group.

The recent update of breast cancer staging by the biologic markers improved the outcome prediction in comparison to prior staging based only on anatomical features of the disease. The validation studies involving the reassessment of the Surveillance, Epidemiology, and End Results (SEER) database (*n* = 209,304, 2010–2014) and the University of Texas MD Anderson Cancer Center database (*n* = 3327, years of treatment 2007–2013) according to 8th edition AJCC manual proved the more accurate prognostic information [189,190].

## 5. Prognostic Biomarkers

### 5.1. Estrogen Receptor

Estrogen receptor (ER) is an important diagnostic determinant since approximately 70–75% of invasive breast carcinomas are characterized by significantly enhanced ER expression [191,192]. Current practice requires the measurement of ER expression on both—primary invasive tumors and recurrent lesions. This procedure is mandatory to provide the selection of those patients who will most benefit from the implementation of the endocrine therapy mainly selective estrogen receptor modulators, pure estrogen receptor downregulators, or third-generation aromatase inhibitors [193]. Even though the diagnosis of altered expression of ER is particularly relevant in terms of the proper therapy selection, ER expression might also constitute a predictive factor—patients with high ER expression usually present significantly better clinical outcomes [194]. A relationship was observed between ER expression and the family history of breast cancer which further facilitates the utility of ER expression as a diagnostic biomarker of breast cancer especially in cases of familial risk [195]. Besides, Konan et al. reported that ERα-36 expression could constitute one of the potential targets of PR-positive cancers and a prognostic marker at the same time [196].

### 5.2. Progesterone Receptor

PR is highly expressed (>50%) in patients with ER-positive while quite rarely in those with ER-negative breast cancer [197]. PR expression is regulated by ER therefore, physiological values of PR inform about the functional ER pathway [197]. However, both ER and PR are abundantly expressed in breast cancer cells and both are considered as diagnostic and prognostic biomarkers of breast cancer (especially ER-positive ones) [198]. Greater PR expression is positively associated with the overall survival, time to recurrence, and time to either treatment failure or progression while lowered PR levels are usually related to a more aggressive course of the disease as well as poorer recurrence and prognosis [199]. Thus, favorable management of breast cancer patients highly depends on the assessment of PR expression. Nevertheless, the predictive value of PR expression still remains controversial [200].

### 5.3. Human Epidermal Growth Factor Receptor 2

The expression of human epidermal growth factor receptor 2 (HER2) accounts for approximately 15–25% of breast cancers and its status is primarily relevant in the choice of proper management with breast cancer patients; HER2 overexpression is one of the earliest events during breast carcinogenesis [201]. Besides, HER2 increases the detection rate of metastatic or recurrent breast cancers from 50% to even more than 80% [202]. Serum HER2 levels are considered to be a promising real-time marker of tumor presence or recurrence [203]. HER2 amplification leads to further overactivation of the pro-oncogenic signaling pathways leading to uncontrolled growth of cancer cells which corresponds with poorer clinical outcomes in the case of HER2-positive cancers [204]. Overexpression of HER2 also correlates with a significantly shorter disease-free period [205] as well as histologic type, pathologic state of cancer, and a number of axillary nodes with metastatic cancerous cells [205].

### 5.4. Antigen Ki-67

The Ki-67 protein is a cellular marker of proliferation and the Ki-67 proliferation index is an excellent marker to provide information about the proliferation of cancerous cells particularly in the case of breast cancer. The proliferative activities determined by Ki-67 reflect the aggressiveness of cancer along with the response to treatment and recurrence time [206]. Thus, Ki-67 is crucial in terms of the choice of the proper treatment therapy and the potential follow-ups due to recurrence. Though, due to several limitations of the analytical validity of Ki-67 immunohistochemistry, Ki-67 expression levels should be considered benevolently in terms of definite treatment decisions. Ki-67 might be considered as a potential prognostic factor as well; according to a meta-analysis of 68 studies involving 12,155 patients, the overexpression of Ki-67 is associated with poorer clinical outcomes of patients [207]. High expression of Ki-67 also reflects poorer survival rates of breast cancer patients [208]. There are speculations whether Ki-67 could be considered as a potential predictive marker, however, such data is still limited and contradictory.

### 5.5. Mib1

Mib1 (antibody against Ki-67) proliferation index remains a reliable diagnostic biomarker of breast cancer, similarly to Ki-67. A decrease in both Mib1 and Ki-67 expression levels is associated with a good response of breast cancer patients to preoperative treatment [209]. Mib1 levels are significantly greater in patients with concomitant p53 mutations [210]. Mib1 assessment might be especially useful in cases of biopsy specimens small in size, inappropriate for neither mitotic index nor S-phase fraction evaluation [211].

### 5.6. E-Cadherin

E-cadherin is a critical protein in the epithelial-mesenchymal transition (EMT); loss of its expression leads to the gradual transformation into mesenchymal phenotype which is further associated with increased risk of metastasis. The utility of E-cadherin as a breast biomarker is yet questionable, however, some research indicated that its expression is potentially associated with several breast cancer characteristics such as tumor size, TNM stage, or lymph node status [212]. Low or even total loss of E-cadherin expression might be potentially useful in the determination of histologic subtype of breast cancer [213,214]. E-cadherin levels do not seem to be promising in terms of patients’ survival rates assessment, however, there are some reports indicating that higher levels of E-cadherin were associated with shorter survival rates in patients with invasive breast carcinoma [213,215]. Lowered E-cadherin expression is positively associated with lymph node metastasis [216].

### 5.7. Circulating Circular RNA

Circulating circular RNAs (circRNAs) belong to the group of non-coding RNA and were quite recently shown to be crucial in terms of several hallmarks of breast carcinogenesis including apoptosis, enhanced proliferation, or increased metastatic potential [217]. One of the most comprehensively described circRNAs, mostly specific to breast cancer include circFBXW7—which was proposed as a potential diagnostic biomarker as well as therapeutic tool for patients with triple-negative breast cancer (TNBC), as well as hsa_circ_0072309 which is abundantly expressed in breast cancer patients and usually associated with poorer survival rates [218]. Has_circ_0001785 is considered to be promising as a diagnostic biomarker of breast cancer [219]. The number of circRNAs dysregulated during breast carcinogenesis is significant; their expression might be either upregulated (e.g., has_circ_103110, circDENND4C) or downregulated (e.g., has_circ_006054, circ-Foxo3) [220]. Besides, specific circRNAs have been reported in different types of breast cancer such as TNBC, HER2-positive, and ER-positive [221]. Recently it was showed that an interaction between circRNAs and micro-RNA—namely in the form of Cx43/has_circ_0077755/miR-182 post-transcriptional axis, might predict breast cancer initiation as well as further prognosis. Cx43 is transmembrane protein responsible for epithelial homeostasis that mediates junction intercellular communication and its loss dysregulates post-transcriptional axes in breast cancer initiation [222].

### 5.8. P53

Loss-of-function mutations in the TP53 (P53) gene have been found in numerous cancer types including osteosarcomas, leukemia, brain tumors, adrenocortical carcinomas, and breast cancers [223,224]. P53 protein is essential for normal cellular homeostasis and genome maintenance by mediating cellular stress responses including cell cycle arrest, apoptosis, DNA repair, and cellular senescence [225]. The silencing mutation of the P53 gene is evident at an early stage of cancer progression. In breast cancer, the prevalence of TP53 mutations is present in approximately 80% of patients with the TNBC and 10% of patients with Luminal A disease [226].

There have been many studies showing the prognostic role of p53 loss-of-function mutation in breast cancer [227,228]. However, the missense mutations may alters p53 properties causing not only a loss of wild-type function, but also acquisition novel activities-gain of function [229]. The IHC status of p53 has been proposed as a specific prognostic factor in TNBC, and a feature that divides TNBC into 2 distinct subgroups: a p53-negative normal breast-like TN subgroup, and a p53-positive basal-like subgroup with worse overall survival [230,231,232]. However, there is not enough evidence to utilize p53 gene mutational status or immunohistochemically measured protein for determining standardized prognosis in patients with breast cancer [233].

### 5.9. MicroRNA

MicroRNAs (miRNA) are a major class of endogenous non-coding RNA molecules (19–25 nucleotides) that have regulatory roles in multiple pathways [234]. Some miRNAs are related to the development, progression, and response of the tumor to therapy [235]. Several studies have investigated abnormally expressed miRNAs as biomarkers in breast cancer tissue samples. According to meta-analysis by Adhami et al. two miRNAs (miRNA-21 and miRNA-210) were upregulated consistently and six miRNAs (miRNA-145, miRNA-139-5p, miRNA-195, miRNA-99a, miRNA-497, and miRNA-205) were downregulated consistently in at least three studies [236].

The miRNA-21 overexpression was observed in TNBC tissues and was associated with enhanced invasion and proliferation of TNBC cells as well as downregulation of the PTEN expression [237]. Similarly, the high expression of miRNA-210 is related to tumor proliferation, invasion, and poor survival rates in breast cancer patients [238,239].

The miRNA-145 is an anti-cancer agent having the property of inhibiting migration and proliferation of breast cancer cells via regulating the TGF-β1 expression [240]. However, the miRNA-145 is downregulated in both plasma and tumors of breast cancer patients [241]. Similarly, miRNA-139-5p and miRNA-195 have tumor suppressor activity in various cancers [242,243].

Nevertheless, further clinical researches focusing on these miRNAs are needed to utilize them as reproducible, disease-specific markers that have a high level of specificity and sensitivity.

### 5.10. Tumor-Associated Macrophages

Macrophages are known for their immunomodulatory effects and they can be divided according to their phenotypes into M1- or M2-like states [244,245]. M1 macrophages secrete IL-12 and tumor necrosis factor with antimicrobial and antitumor effects. M2 macrophages produce cytokines, including IL-10, IL-1 receptor antagonist type II, and IL-1 decoy receptor. Therefore, macrophages with M1-like phenotype have been linked to good disease course while M2-like phenotype has been associated with adverse outcome, potentially through immunosuppression and the promotion of angiogenesis and tumor cell proliferation and invasion [246,247]. In literature, tumor-associated macrophages (TAMs) are associated with M2 macrophages which promote tumor growth and metastasis.

For breast cancer, studies have shown that the density of TAMs is related to hormone receptor status, stage, histologic grade, lymph node metastasis, and vascular invasion [248,249,250,251]. According to meta-analysis conducted by Zhao et al. high density of TAMs was related to overall survival disease-free survival [252].

Conversely, M1 polarized macrophages are linked to favorable prognoses in various cancers [253,254,255]. In breast cancer, the high density of M1-like macrophages predicted improved survival in patients with HER2+ phenotype and may be a potential prognostic marker [256].

However, further studies are needed to clarify the influence of macrophages on breast cancer biology as well as investigate the role of their intratumoral distribution and surface marker selection.

### 5.11. Inflammation-Based Models

The host inflammatory and immune responses in the tumor and its microenvironment are critical components in cancer development and progression [257]. The tumor-induced systemic inflammatory response leads to alterations of peripheral blood white blood cells [258]. Therefore, the relationship between peripheral blood inflammatory cells may serve as an accessible and early method of predicting patient prognosis. Recent studies have reported the predictive role of the inflammatory cell ratios: neutrophil-to-lymphocyte ratio, the lymphocyte-to-monocyte ratio, and the platelet-to-lymphocyte ratio for prognosis in different cancers [258,259,260,261].

#### 5.11.1. The Neutrophil-to-Lymphocyte Ratio (NLR)

In an extensive study on 27,031 cancer patients, Proctor et al. analyzed the prognostic value of NLR and found a significant relationship between NLR and survival in various cancers including breast cancer [262]. There are pieces of evidence of the role of lymphocytes in breast cancer immunosurveillance [263,264]. Opposingly neutrophils suppress the cytolytic activity of lymphocytes, leading to enhanced angiogenesis and tumor growth and progression [265].

Azab et al. first reported that NLR before chemotherapy was an independent factor for long-term mortality and related it to age and tumor size in breast cancer [266]. In a recent meta-analysis by Guo et al., performed on 17,079 individuals, the high NLR level was associated with both poor overall survival as well as disease-free survival for breast cancer patients. Moreover, it was reported that association between NLR and overall survival was stronger in TNBC patients than in HER2-positive ones [267].

#### 5.11.2. Lymphocyte-to-Monocyte Ratio

The association of the lymphocyte-to-monocyte ratio (LMR) with patients’ prognosis has been reported for several cancers [268,269]. As lymphocytes have an antitumor activity by inducing cytotoxic cell death and inhibiting tumor proliferation [270], the monocytes are involved in tumorigenesis, including differentiation into TAMs [246,247,271]. In the tumor microenvironment, cytokines, and free radicals that are secreted by monocytes and macrophages are associated with angiogenesis, tumor cell invasion, and metastasis [271].

A meta-analysis investigating the prognostic effect of LMR showed that low LMR levels are associated with shorter overall survival outcomes in Asian populations, TNBC patients, and patients with non-metastatic and mixed stages [272]. Moreover, high LMR levels are associated with favorable disease-free survival of breast cancer patients under neoadjuvant chemotherapy [273].

#### 5.11.3. Platelet-to-Lymphocyte Ratio (PLR)

A high platelet count has been associated with poor prognosis in several types of cancers [274,275,276]. Platelets contain both pro-inflammatory molecules and cytokines (P-selectin, CD40L, and interleukin (IL)-1, IL-3, and IL-6) and many anti-inflammatory cytokines. Tumor angiogenesis and growth may be stimulated by the secretion of platelet-derived growth factor, vascular endothelial growth factor, transforming growth factor-beta, and platelet factor 4 [277,278,279].

A meta-analysis study investigated the prognostic importance of PLR by analyzing 5542 breast cancer patients. High PLR level was associated with poor prognosis (overall survival and disease-free survival), yet, its prognostic value was not determined for molecular subtypes of breast cancer. Nevertheless, an association was found between PLR and clinicopathological features of the tumor, including stage, lymph node metastasis, and distant metastasis [280]. In the aforementioned meta-analysis, there was a difference in the incidence of high levels of PLR between HER2 statuses [280], while other studies found a difference between hormone ER or PR statuses [281,282].

## 6. Treatment Strategies

### 6.1. Surgery

There are two major types of surgical procedures enabling the removal of breast cancerous tissues and those include (1) breast-conserving surgery (BCS) and (2) mastectomy. BCS—also called partial/segmental mastectomy, lumpectomy, wide local excision, or quadrantectomy—enables the removal of the cancerous tissue with simultaneous preservation of intact breast tissue often combined with plastic surgery technics called oncoplasty. Mastectomy is a complete removal of the breast and is often associated with immediately breast reconstruction. The removal of affected lymph nodes involves sentinel lymph node biopsy (SLNB) and axillary lymph node dissection (ALND). Even though BCS seems to be highly more beneficial for patients, those who were treated with this technique often show a tendency for a further need for a complete mastectomy [283]. However, usage of BCS is mostly related to significantly better cosmetic outcomes, lowered psychological burden of a patient, as well as reduced number of postoperative complications [284]. Guidelines of the European Society for Medical Oncology (ESMO) for patients with early breast cancer make the choice of therapy dependent to tumor size, feasibility of surgery, clinical phenotype, and patient’s willingness to preserve the breast [285].

### 6.2. Chemotherapy

Chemotherapy is a systemic treatment of BC and might be either neoadjuvant or adjuvant. Choosing the most appropriate one is individualized according to the characteristics of the breast tumor; chemotherapy might also be used in the secondary breast cancer. Neoadjuvant chemotherapy is used for locally advanced BC, inflammatory breast cancers, for downstaging large tumors to allow BCS or in small tumors with worse prognostics molecular subtypes (HER2 or TNBC) which can help to identify prognostics and predictive factors of response and can be provided intravenously or orally. Currently, treatment includes a simultaneous application of schemes 2–3 of the following drugs—carboplatin, cyclophosphamide, 5-fluorouracil/capecitabine, taxanes (paclitaxel, docetaxel), and anthracyclines (doxorubicin, epirubicin). The choice of the proper drug is of major importance since different molecular breast cancer subtypes respond differently to preoperative chemotherapy [286]. Preoperative chemotherapy is comparably effective to postoperative chemotherapy [287].

Even though chemotherapy is considered to be effective, its usage very often leads to several side effects including hair loss, nausea/vomiting, diarrhea, mouth sores, fatigue, increased susceptibility to infections, bone marrow supression, combined with leucopenia, anaemia, easier bruising or bleeding; other less frequent side effects include cardiomyopathy, neuropathy, hand-foot syndrome, impaired mental functions. In younger women, disruptions of the menstrual cycle and fertility issues might also appear. Special form of chemotherapy is electrochemotherapy which can be used in patients with breast cancer that has spread to the skin, however, it is still quite uncommon and not available in most clinics.

### 6.3. Radiation Therapy

Radiotherapy is local treatment of BC, typically provided after surgery and/or chemotherapy. It is performed to ensure that all of the cancerous cells remain destroyed, minimizing the possibility of breast cancer recurrence. Further, radiation therapy is favorable in the case of metastatic or unresectable breast cancer [288]. Choice of the type of radiation therapy depends on previous type of surgery or specific clinical situation; most common techniques include breast radiotherapy (always applied after BC), chest-wall radiotherapy (usually after mastectomy), and ‘breast boost’ (a boost of high-dose radiotherapy to the place of tumor bed as a complement of breast radiotherapy after BCS). Regarding breast radiotherapy specifically, several types are distinguished including

(1) intraoperative radiation therapy (IORT)
(2) 3D-conformal radiotherapy (3D-CRT)
(3) intensity-modulated radiotherapy (IMRT)
(4) brachytherapy—which refers to internal radiation in contrast to other above-mentioned techniques.

Irritation and darkening of the skin exposed to radiation, fatigue, and lymphoedema are one of the most common side effects of radiation therapy applied in breast cancer patients. Nonetheless, radiation therapy is significantly associated with the improvement of the overall survival rates of patients and lowered risk of recurrence [289].

### 6.4. Endocrinal (Hormonal) Therapy

Endocrinal therapy might be used either as a neoadjuvant or adjuvant therapy in patients with Luminal–molecular subtype of BC; it is effective in cases of breast cancer recurrence or metastasis. Since the expression of ERs, a very frequent phenomenon in breast cancer patients, its blockage via hormonal therapy is commonly used as one of the potential treatment modalities. Endocrinal therapy aims to lower the estrogen levels or prevents breast cancer cells to be stimulated by estrogen. Drugs that block ERs include selective estrogen receptor modulators (SERMs) (tamoxifen, toremifene) and selective estrogen receptor degraders (SERDs) (fulvestrant) while treatments that aim to lower the estrogen levels include aromatase inhibitors (AIs) (letrozole, anastrazole, exemestane) [290,291]. In the case of pre-menopausal women, ovarian suppression induced by oophorectomy, luteinizing hormone-releasing hormone analogs, or several chemotherapy drugs, are also effective in lowering estrogen levels [292]. However, approximately 50% of hormonoreceptor-positive breast cancer become progressively resistant to hormonal therapy during such treatment [293]. *Endocrinal* therapy combined with chemotherapy is associated with the reduction of mortality rates amongst breast cancer patients [294].

### 6.5. Biological Therapy

Biological therapy (targeted therapy) can be provided at every stage of breast therapy– before surgery as neoadjuvant therapy or after surgery as adjuvant therapy. Biological therapy is quite common in HER2-positive breast cancer patients; major drugs include trastuzumab, pertuzumab, trastuzumab deruxtecan, lapatinib, and neratinib [295,296,297,298,299]. Further, the efficacy of angiogenesis inhibitors such as a recombinant humanized monoclonal anti-VEGF antibody (rhuMAb VEGF) or bevacizumab are continuously investigated [300].

In the case of Luminal, HER2-negative breast cancer, pre-menopausal women more often receive everolimus -TOR inhibitor with exemestane while postmenopausal women often receive CDK 4–6 inhibitor palbociclib or ribociclib simultaneously, combined with hormonal therapy [301,302,303]. Two penultimate drugs along with abemaciclib and everolimus can also be used in HER2-negative and estrogen-positive breast cancer [304,305]. Atezolizumab is approved in triple-negative breast cancer, while denosumab is approved in case of metastasis to the bones [306,307,308].

## 7. Conclusions

In this review, we aimed to summarize and update the current knowledge about breast cancer with an emphasis on its current epidemiology, risk factors, classification, prognostic biomarkers, and available treatment strategies. Since both the morbidity and mortality rates of breast cancer have significantly increased over the past decades, it is an urgent need to provide the most effective prevention taking into account that modifiable risk factors might be crucial in providing the reduction of breast cancer incidents. So far, mammography and sonography is the most common screening test enabling quite an early detection of breast cancer. The continuous search for prognostic biomarkers and targets for the potential biological therapies has significantly contributed to the improvement of management and clinical outcomes of breast cancer patients.

## Figures and Tables

**Table 1:** Modifiable and non-modifiable risk factors of breast cancer.

Non-Modifiable Factors	Modifiable Factors
Female sex	Hormonal replacement therapy
Older age	Diethylstilbestrol
Family history (of breast or ovarian cancer)	Physical activity
Genetic mutations	Overweight/obesity
Race/ethnicity	Alcohol intake
Pregnancy and breastfeeding	Smoking
Menstrual period and menopause	Insufficient vitamin supplementation
Density of breast tissue	Excessive exposure to artificial light
Previous history of breast cancer	Intake of processed food
Non-cancerous breast diseases	Exposure to chemicals
Previous radiation therapy	Other drugs

**Table 2:** Major genes associated with an increased risk of breast cancer occurrence.

Penetration	Gene	Chromosome Location	Associated Syndromes/Disorders	Major Functions	Breast Cancer Risk	Ref.
**High**	*BRCA1*	17q21.31	Breast cancer Ovarian cancer Pancreatic cancer Fanconi anemia	DNA repair Cell cycle control	45–87%	[40]
	*BRCA2*	13q13.1	Breast cancer Ovarian cancer Pancreatic cancer Prostate cancer Fallopian tube cancer Biliary cancer Melanoma Fanconi anemia Glioblastoma Medulloblastoma Wilms tumor	DNA repair Cell cycle control	50–85%	[41]
	*TP53*	17p13.1	Breast cancer Colorectal cancer Hepatocellular carcinoma Pancreatic cancer Nasopharyngeal carcinoma Li-Fraumeni syndrome Osteosarcoma Adrenocortical carcinoma	DNA repair Cell cycle control Induction of apoptosis Induction of senescence Maintenance of cellular metabolism	20–40% (even up to 85%)	[42]
	*CDH1*	16q22.1	Breast cancer Ovarian cancer Endometrial carcinoma Gastric cancer Prostate cancer	Regulation of cellular adhesions Control of the epithelial cells (proliferation and motility)	63–83%	[43]
	*PTEN*	10q23.31	Breast cancer Prostate cancer Autism syndrome Cowden syndrome 1 Lhermitte-Duclos syndrome	Cell cycle control	50–85%	[44]
	*STK11*	19p13.3	Breast cancer Pancreatic cancer Testicular tumor Melanoma Peutz-Jeghers syndrome	Cell cycle control Maintenance of energy homeostasis	32–54%	[45]
**Moderate**	*ATM*	11q22.3	Breast cancer Lymphoma T-cell prolymphocytic leukemia Ataxia-teleangiectasia	DNA repair Cell cycle control	20–60%	[46]
	*PALB2*	16p12.2	Breast cancer Pancreatic cancer Fanconi anemia	DNA repair	33–58%	[47]
	*BRIP1*	17q23.2	Breast cancer Fanconi anemia	Involvement in the *BRCA1* activity	ND	[48]
	*CHEK2*	22q12.1	Breast cancer Li-Fraumeni syndrome Prostate cancer Osteosarcoma	Cell cycle control	20–25%	[49]
	*XRCC2*	7q36.1	Fanconi anemia Premature ovarian failure Spermatogenic failure	DNA repair	ND	[50]
